# Titanium Dioxide Thin Films Produced on FTO Substrate Using the Sol–Gel Process: The Effect of the Dispersant on Optical, Surface and Electrochemical Features

**DOI:** 10.3390/ma16083147

**Published:** 2023-04-16

**Authors:** Vasilica Mihaela Mîndroiu, Andrei Bogdan Stoian, Roberta Irodia, Roxana Trușcă, Eugeniu Vasile

**Affiliations:** Faculty of Chemical Engineering and Biotechnologies, University Politehnica of Bucharest, 1-7 Polizu, 011061 Bucharest, Romania

**Keywords:** TiO_2_ thin films, sol–gel dip-coating technique, FTO substrate

## Abstract

In this study, TiO_2_ thin films formed by dip-coating on an FTO substrate were obtained and characterized using surface, optical and electrochemical techniques. The impact of the dispersant (polyethylene glycol-PEG) on the surface (morphology, wettability, surface energy), optical (band gap and Urbach energy) and electrochemical (charge-transfer resistance, flat band potential) properties were investigated. When PEG was added to the sol–gel solution, the optical gap energy of the resultant films was reduced from 3.25 to 3.12 eV, and the Urbach energy increased from 646 to 709 meV. The dispersant addition in the sol–gel process influences surface features, as evidenced by lower contact-angle values and higher surface energy achieved for a compact film with a homogenous nanoparticle structure and larger crystallinity size. Electrochemical measurements (cycle voltammetry, electrochemical impedance spectroscopy and the Mott–Schottky technique) revealed improved catalytic properties of the TiO_2_ film, due to a higher insertion/extraction rate of protons into the TiO_2_ nanostructure, as well as a decrease in charge-transfer resistance from 418 k to 23.4 k and a decrease in flat band potential from 0.055 eV to −0.019 eV. The obtained TiO_2_ films are a promising alternative for technological applications, due to their advantageous surface, optical and electrochemical features.

## 1. Introduction

Titanium dioxide (TiO_2_) thin films with high specific surface area and narrow pore-size distribution have piqued the interest of industry professionals due to their excellent optical, electrical, and photoelectrochemical properties [1]. TiO_2_ is a nontoxic material with wide band gap in its three crystalline forms: rutile (tetragonal), anatase (tetragonal), and brookite (orthorhombic) [2]. Among these, the anatase phase of TiO_2_ has sparked interest among scientists in a variety of fields, due to its potential use in catalysis and photo-catalysis [3,4] for air and water clearing and also in water splitting [5] and in organic pollutant degradation [6], energy storage and solar energy conversion [7,8], electrochromic and self-cleaning smart windows [9,10] and in biomedical applications [11,12]. The synthesis method, experimental settings, sol composition [13] and polymorph structure are just a few of the variables that affect the properties of TiO_2_ nanoparticles [14,15]. Chemical vapor deposition (CVD) [16], radio frequency (RF) magnetron sputtering (SPS) [17], nebulized spray deposition [9] and the sol–gel procedure [18] are only some of the more common methods currently used to create anatase TiO_2_ thin films. The sol–gel approach is the most appealing of the mentioned methods, due to the simplicity of the required equipment, low processing temperature, good homogeneity, and the ability to achieve multilayer deposition [19] on different substrates, including metals [20], indium-doped tin oxide (ITO) [21] or fluorine-doped tin oxide (FTO) glass [9], depending on the application area.

Thus far, the impact of different conditions on the structural and optical features of TiO_2_ thin films has been the subject of numerous investigations. As an example, F. Zeribi et al. investigated how the number of layers affected the physical characteristics of TiO_2_ that were applied to a glass substrate using the sol–gel spin coating process [18]. N. Barati et al. demonstrated that the withdrawal speed used for the dip-coating process influences the coating thickness, and a drying method for coatings is required to achieve a uniform and nanocrystalline TiO_2_ coating on 316L stainless steel [20]. Other studies investigated the effect of the additive and annealing temperatures on the surface morphology of spin-coated TiO_2_ thin films [22,23,24]. To change the morphology and crystallinity, some complex agents (such as acetylacetone, diethanolamine, polyethylene glycol, Lauryl lactyl lactate) [25,26] or surfactants (such as cationic-sodium dodecyl sulphate (SDS), cetyltrimethylammonium bromide (CTAB), and non-ionic (TritonX-100)) [27] were added. As a result, particle agglomeration was reduced and TiO_2_ coating stability and photocatalytic efficiency were improved. Moreover, when two or more distinct surfactants were introduced to the mixture simultaneously, the morphology of TiO_2_ changed substantially, adopting spherical and three-dimensional forms because of surfactant coordination [27].

Efforts are still being made to obtain adherent and continuous TiO_2_ films on FTO or ITO glass electrodes with improved optical properties in the visible range, as well as improved electrochemical and electrochromic properties, with the goal of using these electrodes in cutting-edge applications, such as smart windows, which are of great interest today, given global warming and the energy crisis.

This study determines the optimal number of dip-coating layers and the effect of the dispersant (PEG) added to the sol–gel precursor solution on the optical (as reduced band gap and increased Urbach energies), electrochemical (as high insertion/extraction rate of protons, decrease in charge-transfer resistance, and flat band potential) and surface (nanomorphology, crystallinity, decreased wettability and high surface energy) properties of titanium dioxide thin films obtained on FTO.

The results of this research will help to develop a practical procedure to create pseudo-capacitive electrodes for technological applications by correlating the surface morphology, vibrational, and optical properties of synthesized TiO_2_ thin films on FTO substrates with electrochemical properties.

## 2. Experimental Techniques

### 2.1. Materials

Titanium butoxide (Ti(OBu)_4_ (Bu = CH_2_CH_2_CH_2_CH_3_)), anhydrous ethanol (C₂H₆O), acetic acid (CH_3_COOH), polyethylene glycol 8000 (PEG), sulphuric acid (H_2_SO_4_) (Alfa Aesar, Haverhill, MA, USA), ≥99.5% ethylene glycol (Honeywell, Charlotte, NC, USA), acetyl acetone (CH₃COCH₂COCH₃) (Sigma Aldrich, Saint Louis, MO, USA), dimethyl sulfoxide (DMSO) (Carlo Erba Reagents, Cornaredo, MI, Italy) and distilled water were used as reagents in the present work. The TiO_2_ films with different thicknesses were deposited on fluorine-doped tin oxide (FTO; R_sh_ = 15 Ω/sq; 2.5 × 5 cm^2^, 1.3 mm thickness, Solaronix, Aubonne, Switzerland) coated glass substrates.

### 2.2. Preparation of TiO_2_ Thin Films on FTO Substrate by Dip-Coating Method

The sol–gel precursor solution (Solution 1) was created by mixing together 8 mL Ti(OBu)_4_, (as alkoxide), 80 mL ethanol (as solvent), 8 mL acetylacetone (as catalyst), 8 mL CH_3_COOH (as pH adjuster, to 6) and 8 mL of water [18,28]. A clear solution was obtained after 3 h of stirring the mixture at 50 °C, which was further aged for 24 h at room temperature. The FTO substrates were cleaned with ethanol, acetone, and distilled water for ten minutes each, then dried in air.

The precursor solution was set up by dip-coating onto the surfaces after they had been cleaned using a KSV NIMA Dip-Coater (Biolin Scientific, Västra Frölunda, Sweden) using the following parameters: immersion speed 3 cm/min, immersion time 30 s and lifting speed 10 cm/min. The resulting film was dried in an oven at 200 °C for 10 min after each deposition cycle for the organic solvent evaporation. To obtain films with different thicknesses, the deposition procedure was repeated 2, 4, 6 and 8 times. The names of the samples obtained by this method are listed as “P_number layer_”. Then, the obtained films were investigated to determine the ideal number of layers needed to generate a TiO_2_ film with a lower band gap energy and higher Urbach energy.

After determining the optimal number of TiO_2_ layers, we investigated how the presence of a dispersant in the precursor solution influences the nanomorphology of the material, electrochemical and optical properties of the resulting TiO_2_ film. In order to accomplish this, a second solution identified as Solution 2 was made by continuously stirring Solution 1 for a period of two hours, until a specific amount of PEG was dissolved [29]. PEG promotes nanopore formation and reduces TiO_2_ nanoparticle aggregation [30]. The names of the samples that were obtained using this method are identified as follows: “P_number layer_PEG_”.

All films obtained from both solutions were annealed in an electric furnace for two hours at 450 °C.

**Figure 1:**
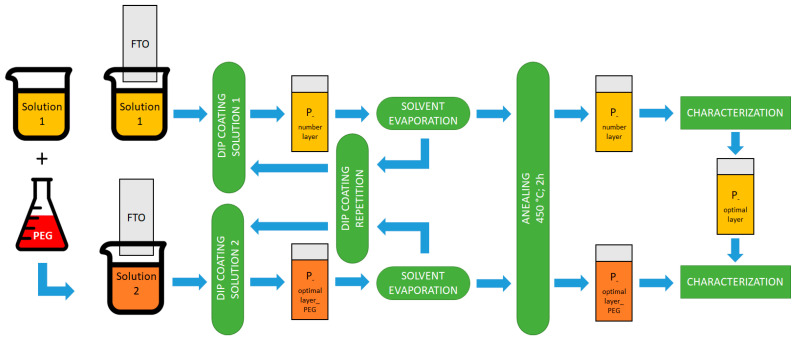
A simplified schematic of the procedure followed to obtain the TiO_2_ thin films on FTO substrate by dip-coating method.

The stages involved in TiO_2_ thin film production are depicted in Figure 1.

### 2.3. Characterization Methods

#### 2.3.1. The Optical Properties

The band gap energy, transmittance and Urbach energy of TiO_2_ films obtained on FTO from Solution 1 and Solution 2, were calculated taking UV–Vis light transmittance measurements in the wavelength range of 300–800 nm, using a Perkin Elmer Lambda (Waltham, MA, USA) 650/850/950 UV/Vis spectrophotometer. These studies were carried out three times each, highlighting a minimum statistical and standard deviation in the data.

#### 2.3.2. Physicochemical Characterization

The crystal structure of the TiO_2_ films was examined with the Panalytical (Malvern, UK) X’Pert Pro MPD X-ray diffractometer, using a CuK α X-ray beam (wavelength 1.54065 Å) and Bragg–Brentano geometry. The effects of the dispersant present in sol–gel dip-coating solution on the microstructure and morphology of the TiO_2_ thin films were investigated using the QUANTA INSPECT F50 (FEI Company, Eindhoven, The Netherlands) scanning electron microscope equipped with a field emission gun (FEG) with a resolution of 1.2 nm and an energy dispersive X-ray spectrometer (EDS) with MnK resolution of 133 eV. The size of the particles were determined using Image J software; at least 10 measurements were performed on SEM images and then the average and standard deviation were calculated. The surface analysis and roughness evaluation were performed using an atomic force microscope (AFM) (APE Research, Trieste, Italy), in contact mode. Structural and chemical bonding of TiO_2_ films were studied via infrared spectroscopy using a Perkin Elmer (Waltham, MA, USA) Spectrum 100 FT-IR spectrometer in the attenuated total reflection mode (ATR). ATR/FT-IR spectra were recorded in the 4000 cm^−1^–600 cm^−1^ domain, representing an average of 4 scans collected at a resolution factor of 4 cm^−1^. The contact angle measurements completed the surface characterization, quantifying the wettability of a solid surface by a liquid and calculating the surface free energy. Thus, the sessile drop technique was used with three different solvents: distilled water, Ethylene Glycol (EG), and dimethyl sulfoxide (DMSO) to determine the contact angle using an optical contact angle measuring instrument (Contact Angle Meter—KSV Instruments CAM 100 equipment) (Biolin Scientific, Västra Frölunda, Sweden).

#### 2.3.3. Testing of Electrochemical Properties

Cyclic voltammetry (CV), electrochemical impedance spectroscopy (EIS) and Mott–Schottky measurements were carried out from −0.6 V to +0.2 V domain range in 0.5 M H_2_SO_4_ aqueous solution, to investigate the electrochemical stability and the interfacial charge-separation efficiencies of the TiO_2_ film as a function of the dispersant presence in sol–gel dip-coating solution. The procedures below were carried out using a three-electrode electrochemical cell: the obtained electrodes were the working electrodes Ag/AgCl, KCl sat. and Pt mesh as reference and counter electrodes, respectively. An Autolab (PGSTAT 204) potentiostat/galvanostat from Metrohm (Herisau, Switserland) system was used to control the parameters as well as collect data.

## 3. Results and Discussions

### 3.1. Determining the Optimum Number of Dip-Coatings Required to Deposit the TiO_2_ Film on the FTO Substrate with the Lowest Band-Gap and Highest Urbach-Energy Values

In the research carried out on the mechanism of thin film growth, three distinct phases of film creation are typically identified and discussed: (1) nucleation, (2) cross linking and (3) vertical growth [18]. The substrate temperature determines the onset and rate of the first two phases, whereas the amount of layer deposition, or film thickness, governs the vertical growth phase. Hence, the source of surface roughness and agglomeration and clusters on the top surface may be traced back to the vertical growth, also known as columnar growth. Thus, the number of layers used for TiO_2_ dip-coating deposition onto the FTO substrate influences surface properties such as roughness and optical features.

**Figure 2:**
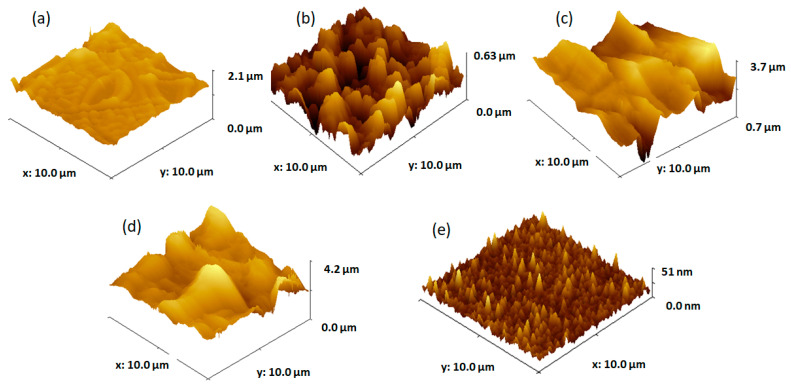
AFM images of TiO_2_ films obtained from solution 1 by (**a**) two dip-coating cycles, (**b**) four dip-coating cycles, (**c**) six dip-coating cycles, (**d**) eight dip-coating cycles, and (**e**) FTO substrate.

According to the AFM image in Figure 2a, the TiO_2_ film produced by two cycles of dip-coating exhibited both tubular and palletized morphologies. The tube dimensions are around 3–5 µm in length and 0.65 µm in width and the pallet constructions have side lengths of 1.4 µm and 0.7 µm. The structures’ heights range from 100 to 200 nm.

The topography of the formed film by four dip-coating cycles on the FTO substrate (Figure 2b) shows that the FTO substrate is completely covered by intricate TiO_2_ structures. The TiO_2_ structures are irregular clusters of around 1–2 µm in length and 200–400 nm in height. The TiO_2_ films formed by six and eight cycles of dip-coating deposition on FTO (Figure 2c,d) completely cover the substrate with irregular TiO_2_ dome-like structures of 3 µm in width and heights ranging from 400 to 700 nm. The topography of the FTO substrate on which the TiO_2_ films were deposited by dip-coating (Figure 2e) reveals that the FTO (fluorine-doped tin oxide) particles are granular in appearance and uniformly cover the glass substrate, with grain diameters ranging from 10 to 50 nm and heights from 10 to 25 nm.

The thickness and roughness of the TiO_2_ films obtained by various dip-coating deposition cycles on the FTO substrate are shown in Table 1.

The TiO_2_ film deposition on the FTO substrates from Solution 1 results in thick layers, with thicknesses ranging from 0.674 µm for two layers to 1.945 µm for eight layers. Once many layers are deposited onto the substrate, the thickness increases as reported in the literature [18,31,32], and microcracks begin to emerge on the surface (as seen in AFM pictures corresponding to six and eight layers). Findings from other studies suggest that internal stresses are generated as the density of nucleation centers decreases with layer thickness [33,34], leading to increased surface microcracks [35]. We expect all these factors to affect the films’ optical characteristics of samples with the most layers deposited.

**Figure 3:**
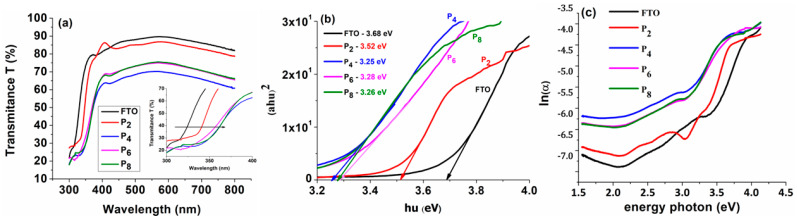
(**a**) Optical transmittance; (**b**) plot of (αhν)^2^ versus hν; and (**c**) ln(α) versus photon energy plot for TiO_2_ thin films deposited on an FTO substrate from solution 1, using different number of dip-coating layers.

The optical transmittance spectra of TiO_2_ thin films deposited from Solution 1 on the FTO substrate in the wavelength range of 300–800 nm are shown in Figure 3a as a function of the number of dip-coating layers.

The inset of Figure 3a shows that once the number of layers increases, the edge of absorption shifts to longer wavelengths as time passes, indicating that the band energy of the films formed on the FTO substrate is reducing. Furthermore, increasing the number of layers of the film makes it thicker and reduces its transmittance. The electronic transition between the TiO_2_ valence band and conduction band induces fundamental light absorption in the TiO_2_ thin films, as shown in the transmittance spectrum (Figure 3a) at 407 nm [36]. In addition, for all obtained films, a high transmission region (over 60%) was found in the visible zone (500–800 nm), indicating anti-reflection and UV-protective properties [37].

Plotting curves between (αhν)^2^ and (hν) yields the optical energy of the band gap of the TiO_2_ film produced on the FTO substrates after two, four, six, and eight dip-coating cycles (Figure 3b). The band gap energy is determined using Tauc’s equation with the following relationship [38]:

(αhν) = A(hν − E_g_) (1)

where E_g_ is the bandgap energy, A is a constant, hν is the energy of the incident photon and α is the absorption coefficient [39].

The band gap value decreases with the increasing number of dip-coating deposition cycles, remaining nearly constant after four deposition cycles, at around 3.26 eV ± 0.2 eV.

Defects in the film lattice in the optical band gap region are represented by the Urbach energy (band tail width), (E_u_), in Figure 3c. The Urbach tail, or absorption tail, can be traced back to flaws in the forbidden band region. The following formulas were used to calculate the Urbach energy from the absorption spectrum [40]:

(2) $$\alpha =\alpha_{0}exp\left(\frac{h\upsilon }{E_{u}}\right)\;\mathrm{and}\;E_{u}={\left(\frac{d[\alpha h\upsilon ]}{d[h\upsilon ]}\right)}^{-1}$$

where α_0_ is a constant and E_u_ is the Urbach energy.

The Urbach energy is estimated from the graph ln(α) = f (photon energy) (Figure 3c): E_u_ = 1/Slope. Table 2 displays the computed band gap energy and Urbach energy of the TiO_2_ thin films after two, four, six, and eight dip-coating cycles.

The band gap energy (E_g_) is found to decrease from 3.52 eV to 3.25 eV as the number of layers increases, while the Urbach energy increases from 310 meV to 646 meV. Based on the AFM results, it can be observed that, due to the appearance of many microcracks in P_6_ and P_8_, the band gap slowly increased and Urbach energy decreased, compared with sample P_4_, which presented a uniform compact film.

These findings are consistent with previous research [41,42,43]. Band gap energy values of TiO_2_ thin layers formed on the glass substrate by sol–gel spin coating were calculated to be between 3.67 and 3.52 eV in earlier research [18]; however, the obtained band gap energy values range from 3.52 to 3.25 eV of the TiO_2_ coated by sol–gel dip-coating on the FTO substrate.

**Figure 4:**
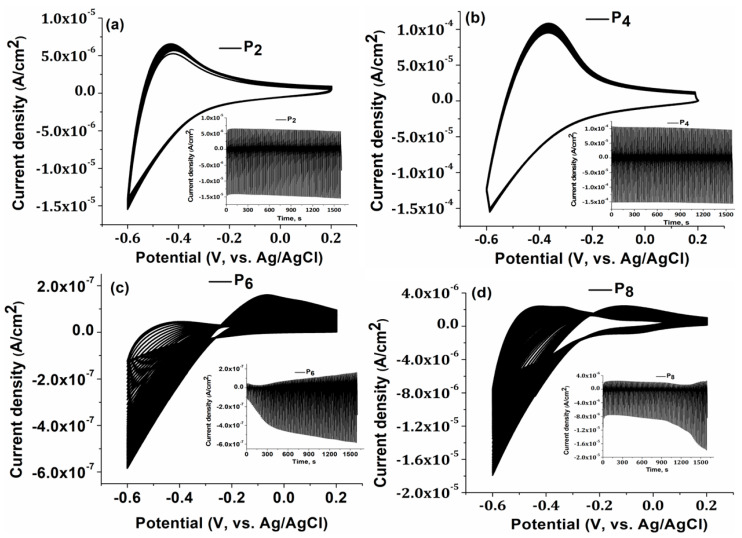
Cycle Voltammetry for (**a**) P_2_; (**b**) P_4_; (**c**) P_6_; (**d**) P_8_ between −0.6 V and 0.2 V vs. Ag/AgCl at 50 mV s^−1^, 100 cycles, in 0.5 M H_2_SO_4_ aqueous solution.

Furthermore, cyclic voltammetry (CV) was performed to examine the relationship between the number of layers and the adherence of the obtained TiO_2_ layers on the FTO substrate (Figure 4). Thus, 100 CV cycles were recorded for all samples, to determine the electrochemical stability of the films on the FTO substrate. Results are illustrated in Figure 4. As the number of CV cycles grew, the corresponding constant current values for P_4_ indicated (Figure 4b) a strong bond on the substrate and good electrochemical stability [44].

The greater the film’s electrochemical stability, the better it adheres to the substrate. Samples of both types, P_2_ and P_4_, are stable, with P_4_ showing a more capacitive behavior. In the samples of P_6_ and P_8_, the results changed substantially after almost 20 CV cycles. Microcracks in samples P_6_ and P_8_ cause an unstable CV signal and a higher optical transmittance compared to sample P_4_, as shown by a correlation between the CV test results, the AFM results, and the optical properties.

This phenomenon keeps these films unstable even as they increase in thickness and develop microcracks. Electrolytes infiltrate through microcracks between the films and the substrate, causing the current to fluctuate as the CV cycles increase.

Based on the current experimental results, it has been concluded that four-cycle dip-coating is the ideal approach for depositing TiO_2_ multilayers onto FTO substrates, because it has the best optical properties and because it presents electrochemical stability.

### 3.2. Evaluating the Impact of the PEG Presence in the Sol–Gel Precursor Solution on the Morphological, Optical and Electrochemical Properties of TiO_2_ Films

#### 3.2.1. Surface Characterization

Given the band gap and Urbach energy results, from which we have determined that the samples with four TiO_2_ layers conferred the best results, henceforth, all the presented data represent the results obtained for the samples obtained after four dip-coating cycles in Solution 1 and in Solution 2.

**Figure 5:**
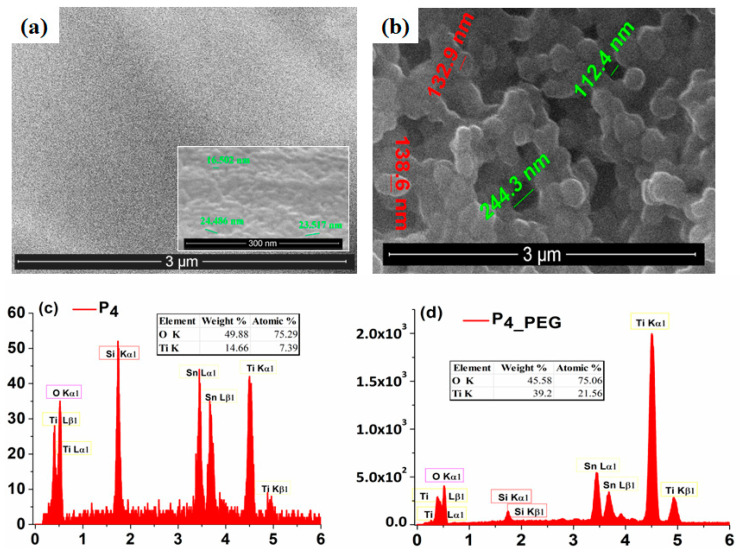
SEM images of (**a**) P_4_ and (**b**) P_4_PEG_. EDAX analysis of (**c**) P_4_ and (**d**) P_4_PEG_.

The SEM image (Figure 5a) obtained for the surface of the P_4_ film indicates a compact and uniform TiO_2_ thin layer with a homogeneous distribution of nanoparticles with an average size of around 23 nm (±2.93), which covers the whole FTO surface.

Conversely, the morphology of the P_4_PEG_ films (Figure 5b) shows the FTO substrate covered by a continuous TiO_2_ film with a homogeneous distribution of nanoparticles with an average size of around 100 nm (±6.85). As in the literature [30], the addition of PEG in the coating solution promoted the growth of a nanostructured film and enhanced the production of nanoparticles.

These SEM results demonstrate that the dispersant can modify the size and shape of TiO_2_ nanoparticles deposited on the FTO substrate by the dip-coating method.

To confirm the presence of both Ti and O in the films obtained from both solutions, the EDX spectra (Figure 5c,d) was performed and recorded. Oxygen is present in nearly equal amounts in both films, while Ti is more abundant in the P_4_PEG_ film.

**Figure 6:**
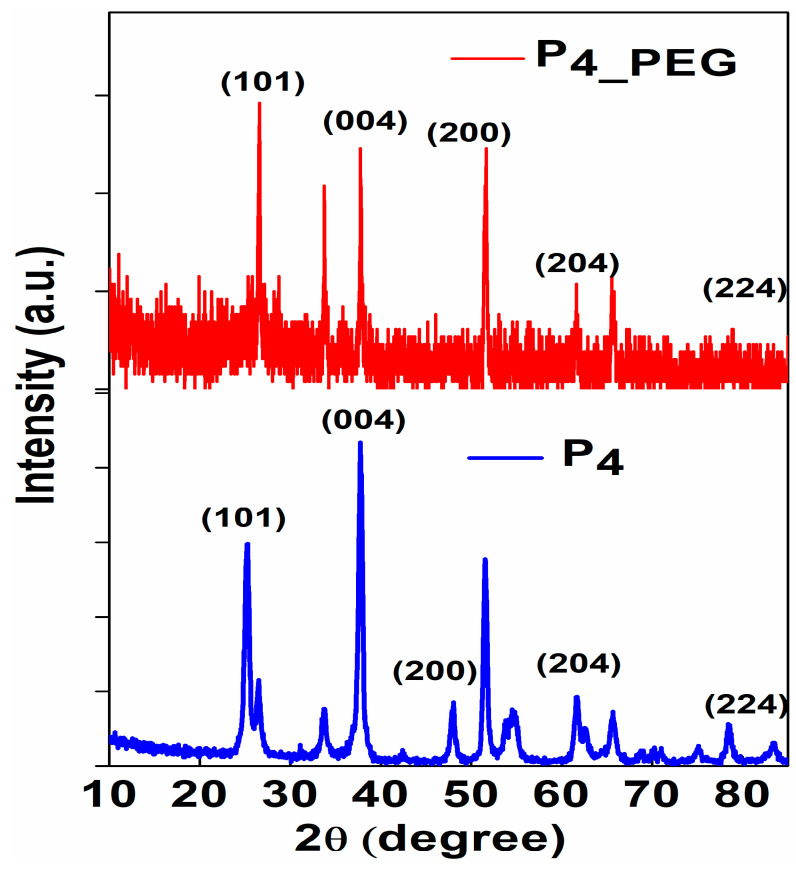
The X-ray diffraction (XRD) patterns of P_4_ and P_4_PEG_ thin films dip-coated on FTO substrate.

The crystalline phase and grain size of the films dip-coated on FTO were investigated using X-ray diffraction patterns (Figure 6).

The characteristic peaks at 26.50° and 26.56°; 37.78° and 37.81°; 48.03° and 47.93°; 62.70° and 61.55°; and 83.48° and 84.33°, corresponding to the (101); (004); (200); (204) and (224) planes of TiO_2_ anatase, respectively, were observed in both the P_4_ and P_4_PEG_ thin films [45,46]. All the samples exhibited a majority crystallite structure of the TiO_2_ anatase phase, and the rutile phase was observed at 53.72° and 51.56°, corresponding to the (105) plane, and at 55.10° and 54.54°, corresponding to the (211) plane, for both obtained films.

The average crystallite size (*D*) of the samples was calculated using the Debye–Scherrer equation [18]:

(3) $$D=\frac{0.94\lambda }{\beta \cos\theta }$$

where *θ* is the Bragg diffraction angle, *λ* is the light wavelength utilized for diffraction, equal to 1.54065 Å, and *β* is the full width at half maxima (FWHM) of the diffraction peak, in radians.

The following relationships were also used to calculate the lattice strain (ε) [47] and dislocation density (δ) [48]:

(4) $$\varepsilon =\frac{\beta }{4\tan\left(\theta \right)}\;and\;\delta =\frac{1}{D^{2}}$$

In Table 3, the variation of crystallite size, the lattice strain and dislocation density are presented.

PEG in solution 2 increases the crystallite size while decreasing the strain, and it is expected to influence optical properties in relation to the band gap’s low energy [18]. This could be due to the relatively easy decomposition of PEG 8000’s high molecular chains during thermal treatment at 450 °C, resulting in the collective fusion of small crystallites into nanoparticle aggregates [15].

**Figure 7:**
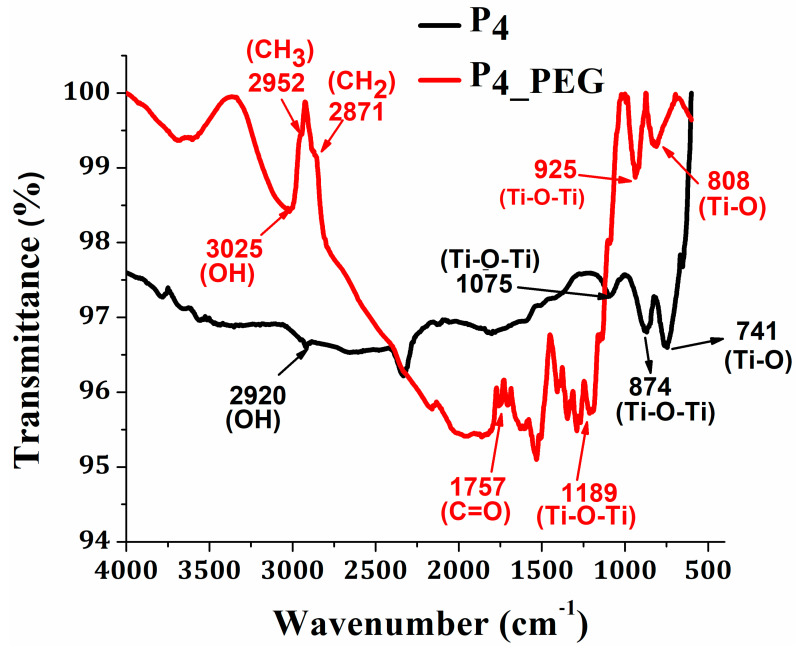
FTIR spectra of TiO_2_ thin films obtained from sol–gel solution with/without PEG.

Furthermore, the ATR/FT-IR spectra of the TiO_2_ thin films prepared from solution 1 without a dispersant and solution 2 with PEG were examined using infrared spectroscopy to determine the type of structural and chemical bonding of TiO_2_ (Figure 7). In both spectra, peaks in the 2920 cm^−1^ and 3025 cm^−1^ regions [18,49] are observed and are associated with the stretching vibration of hydroxyl groups. The absorption peaks at 2952 cm^−1^, 2871 cm^−1^, and 1757 cm^−1^ correspond to the stretching vibrations of the CH_3_, CH_2_, and C=O groups, respectively, of the PEG from the P_4_PEG_ film [50]. The Ti-O-Ti bonds in the TiO_2_ nanoparticles’ stretching vibration mode are responsible for the absorption bands at 1075 cm^−1^ and 874 cm^−1^, which correspond to P_4_ [18,51]. The film with PEG presents the same Ti-O-Ti stretching vibration mode, but it is shifted to 1189 cm^−1^ and 925 cm^−1^ for the P_4_PEG_ film. The stretching vibration of the Ti-O groups in the anatase phase [18,52] was found at 741 cm^−1^ and 662 cm^−1^ for P_4_ and at 808 cm^−1^ and 601 cm^−1^ for P_4_PEG_ [30].

As expected, results from Table 4 indicate that thin TiO_2_ films displayed low contact angle values due to the hydrophilic nature of the mixture components coated on the substrate. The PEG presence in the sol–gel solution influences the wettability of the obtained TiO_2_ thin film; the contact angle value (CA = 16°) of the film with a dispersant is lower than the one corresponding to the film without a dispersant (CA = 26°).

Surface free energy, which is a result of film surface defects [53], is important for the pseudo-capacitive processes that occur in many applications such as smart windows. The results of this study indicate that the TiO_2_ films’ surface free energy varies with the addition of the dispersant. The Owens–Wendt–Rabel–Kaelble (OWRK) model was successfully applied to calculate the surface free energy [54]. In terms of contact angles, the lower the surface energy, the greater the contact angles.

#### 3.2.2. Electrochemical Characterization

**Figure 8:**
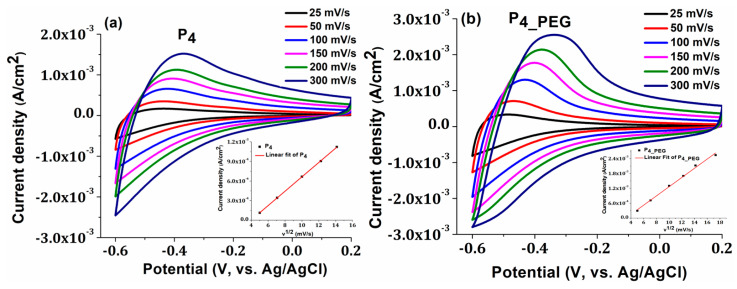
The cyclic voltammograms of (**a**) P_4_ and (**b**) P_4_PEG_ films on FTO at various scan rates, with an insert displaying the plot of oxidation peak current densities vs. square root of potential scan rate.

The electrochemical behavior of TiO_2_ films in 0.5 M H_2_SO_4_ solution was investigated during the cycling of applied potentials ranging from −0.6 to +0.2 V at different scan rates of 25, 50, 100, 150, 200, and 300 mV s^−1^. Both obtained films exhibited an oxidation peak near to the Fermi level of anatase TiO^2^ at −0.5 V (Figure 8) [9]. At rates greater than 50 mV/s, the oxidation peak shifts to less-negative potential values of −0.4 V, due to the oxidation of Ti^3+^ to Ti^4+^, because of the deintercalation of H^+^ ions and electrons from TiO_2_ nanostructures via the reaction outlined below [55]:

TiO_2_ + xe^−^ + xH^+^ → TiOOH

A shift in peak potentials toward higher positive values indicates a desirable behavior for the utilization of these electrodes in technological applications with catalytic behavior, such as smart windows.

Moreover, the capacitive behavior of the TiO_2_ films obtained in the presence of the dispersant is more prominent, and the current density increases, indicating a more conductive character than the film obtained without PEG. Straight lines are obtained when the peak value of the oxidation current is plotted against the square root of the scan speed for both samples (inset Figures), indicating that the insertion and extraction of H^+^ ions is rapid and that proton diffusion in the TiO_2_ nanostructure controls the rate-determining step of the redox reaction. Due to the nanocrystalline form of the grains, according to the SEM image (Figure 5b), the diffusion and charge-transfer process of H^+^ ions in the TiO_2_ thin film may be facilitated, which may explain why greater charge densities were reported in the P_4_PEG_ thin film [9].

**Figure 9:**
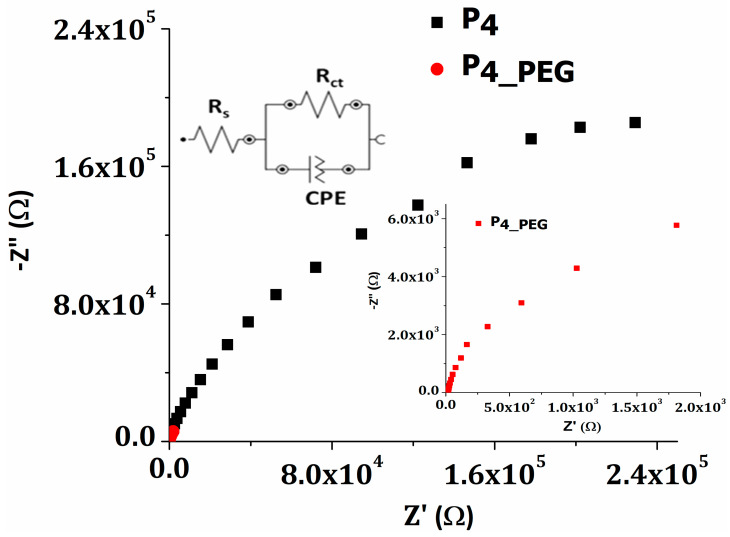
Nyquist plots for P_4_ and P_4_PEG_ films.

To investigate the charge-transfer kinetics and mass transport behavior of the films developed on the FTO substrate, electrochemical impedance spectroscopy (EIS) analysis was performed. The EIS measurements were made in a frequency range of 10 mHz to 100 kHz. Figure 9 shows the Nyquist plots of the P_4_ and P_4_PEG_ thin films measured at free potential voltage in 0.5 M H_2_SO_4_ solution.

To fit the data, the Randles equivalent circuit model was used, as can be seen in the inset of Figure 9. The equivalent circuit consists of solution resistance (R_s_), charge-transfer resistance (R_ct_) across the developed electrodes/H_2_SO_4_ electrolyte interface, and a constant phase element (CPE) which represent a double-layer capacitance at the electrode surface. Table 5 shows the electric parameters from the EIS experimental data fitted with the proposed equivalent circuit.

The charge-transfer resistance (R_ct_) of the P_4_ and P_4_PEG_ films deposited on the FTO by dip-coating is 418 kΩ and 23.4 kΩ, respectively. As previously noted, the higher surface free energy, as well as the smaller band gap and higher Urbach energy of the film, produce low charge-transfer resistance, which provides less resistance for ion transport and the slowest recombination rate. When TiO_2_ was doped with PEG, the density of the nanoparticles in the film increased, leading to higher conductivities. PEG is a well-known long-chain surfactant that helps prevent TiO_2_ agglomeration during the dip-coating deposition process [56]. The higher value obtained for the P_4_ film, indicating poor charge-transfer characteristics, is improved by adding PEG to the sol–gel precursor solution. The double-layer capacity of the P_4_PEG_ film is three orders of magnitude greater than that of the TiO_2_ film prepared from solution 1. Both films exhibited pseudo-capacitive behavior as a result of *n* values ranging from 0.8 to 1 [57].

The Mott–Schottky (M-S) Equation (5) was used for further analysis of the impedance data and is defined as follows:

(5) $$\frac{1}{C^{2}}=\left(\frac{2}{q\varepsilon \varepsilon_{0N_{D}}}\right)\left(E-E_{fb}-\frac{kT}{q}\right)$$

where *C* is the capacity of the space charge layer, *q* is the elementary charge, *ε*_0_ is the vacuum permittivity, *ε* is the dielectric constant, *N_D_* is the concentration of donors, *E* is the applied external bias, *E_fb_* is the flat band potential, *k* is Boltzmann’s constant, and *T* is the absolute temperature.

Flat band potential (*E_fb_*) is determined by extrapolating to 1/*C*^2^ = 0, which is dependent on the recombination process and interface charge-transfer [58], whereas the slope of the Mott–Schottky plot indicates the doping level (*N_D_*). Indirect electron tunnelling through a semiconductor is a well-known result of a band gap with a large concentration of numerous donor levels.

**Figure 10:**
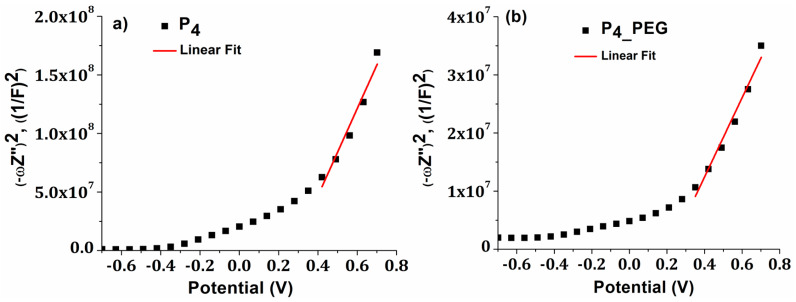
Mott–Schotky (M-S) plots for (**a**) P_4_ and (**b**) P_4_PEG_ films.

Each P_4_ and P_4_PEG_ film displayed a positive slope on the Mott–Schottky graphs, as is typical for n-type semiconductors [59], (Figure 10).

E_fb_ is typically regarded as the conduction band potential (CB) for n-type semiconductors [59]. The calculated value of E_fb_ vs. NHnE (normal hydrogen electrode) for P_4_ and P_4_PEG_ is 0.055 eV and −0.019 eV, respectively. These results imply a slower recombination rate for film formed in the presence of a dispersant, which agrees with the reduction in charge-transfer resistance measured by EIS. The negative shift in the flat-band potential for P_4_PEG_ suggests that the energy barrier for interfacial electron transport is reduced, resulting in a lower charge-transfer resistance [60]. Moreover, the doping level (N_D_) for P_4_ and P_4_PEG_ are 6.5 × 10^19^ cm^−3^ and 2.01 × 10^20^ cm^−3^, respectively, correlating with the variation in Urbach energy values. We may conclude that adding PEG to the sol–gel precursor solution induces a shift in the flat band potential of TiO_2_ to negative values, indicating improved catalytic capabilities and a higher doping level.

#### 3.2.3. Optical Properties—Bandgap and Urbach Energies

Using Tauc’s equation (Equation (1)) and based on UV–VIS data, the band gap energy was determined.

**Figure 11:**
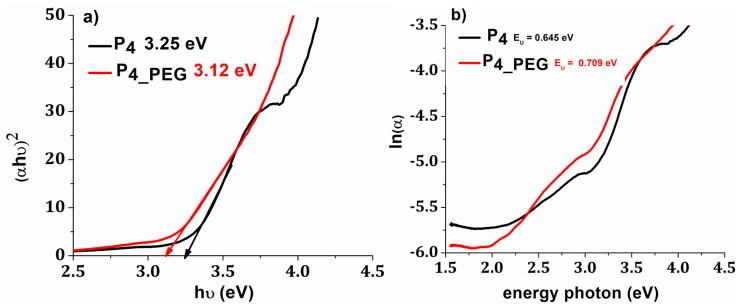
(**a**) plot of (αhν)^2^ versus hν; and (**b**) plot of ln(α) versus photon energy.

The band gap (E_g_) values for P_4_ were reduced from 3.25 eV to 3.12 eV for P_4_PEG_, indicating that the films are more conductive (Figure 11). In addition, for thin films made with a dispersant, the Urbach energy increased from 0.645 eV to 0.709 eV, with these values correlating with the increased surface free energy (Table 4). More structural defects, as shown by the Urbach energy value, can be induced by the delocalization of molecular orbitals caused by the simple breakdown of long interweaving PEG molecule chains, resulting in greater absorption at longer wavelengths and a decrease in band gap value [53,61].

**Figure 12:**
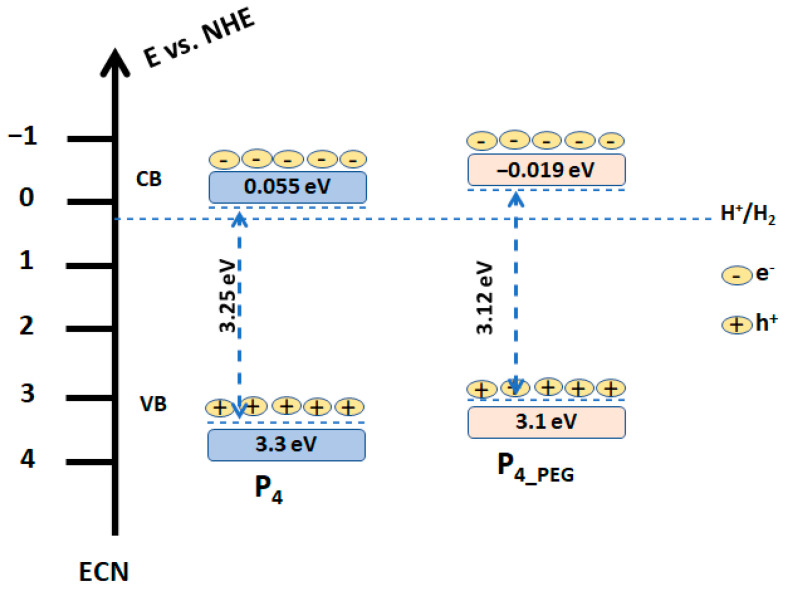
Schematic diagram of the band gap of the P_4_ and P_4_PEG_.

The structure diagrams for the obtained films based on the measured M–S flat band potential and band gap from the UV–VIS data are shown in Figure 12. Based on the measured CB and E_g_, the valence band (VB) energy of the films deposited on the FTO was computed.

As can be seen, P_4_ VB is 3.3 eV, while P_4_PEG_ VB is 3.1 eV. When compared to P_4_ and P_4_PEG_, the VB is reduced by 0.2 eV. This suggested that the presence of PEG in solution 2 generates electrons on the surface of the film coated on the FTO substrate and improves conductivity, due to the well-controlled grain of the TiO_2_ (as depicted in Figure 5b), with greater crystallinity, as observed in the XRD data (Figure 6).

Based on these findings, the P_4_PEG_/FTO electrode can be employed in a variety of applications, including smart windows and photocatalysts for solar cells or water splitting, where pseudo-capacitive behavior is desired.

## 4. Conclusions and Perspectives

The surface characteristics of the TiO_2_ film deposited on the FTO substrate are enhanced by the incorporation of PEG into the sol–gel precursor solution used for the dip-coated deposition process. This results in a TiO_2_ film with increased free energy, as well as a more compact structure and nanoparticles with a higher degree of crystallization. The optical and electrochemical properties are affected by these surface features. With a higher insertion/extraction rate of protons into the P_4_PEG_ nanostructure, the band gap is decreased from 3.25 to 3.12 eV, and the Urbach energy increases from 646 to 709 meV. The charge-transfer resistance is decreased from 418 kΩ to 23.4 kΩ, and the flat band potential is decreased from 0.44 V to −0.019 V, indicating improved catalytic properties of the final TiO_2_ film. These features make P_4_PEG_ film a competitive option for technical uses such as smart windows, solar cells, photocatalysts and gas sensors.

Our long-term goal is to produce a pseudo-capacitive electrochromic electrode using this developed P_4_PEG_ film as a template, which will be employed in the production of smart windows.

## Figures and Tables

**Table 1:** The thickness and roughness of TiO_2_ films.

Number of Layers	Film Thickness, µm	Roughness Average (R_a_), µm
2	0.674 ± 0.057	0.667 ± 0.074
4	1.346 ± 0.095	0.432 ± 0.065
6	1.881 ± 0.051	0.412 ± 0.038
8	1.945 ± 0.055	0.259 ± 0.045
FTO	-	0.004 ± 0.002

**Table 2:** Band gap and Urbach energies for TiO_2_ thin films deposited on FTO substrate using sol–gel dip-coating at varying cycle numbers.

Number of Layers	Band Gap Energy, E_g_ (eV)	Urbach Energy, E_u_ (eV)
2	3.52 ± 0.018 3.25 ± 0.013 3.28 ± 0.017 3.26 ± 0.009 3.68 ± 0.018	0.3104 ± 0.005 0.6459 ± 0.012 0.6142 ± 0.011 0.61337 ± 0.013 0.2787 ± 0.013
4		
6		
8		
FTO		

**Table 3:** Crystallite sizes, strain, and dislocation density values of P_4_ and P_4_PEG_ thin films from XRD results.

Samples	Peak (hkl)	2-Theta	FWHM (β)	D (nm)	$\varepsilon \;\times \;10^{-4}$	$\delta \times 10^{15}$ (m^−2^)
**P_4_**	(101)	26.50	0.384	22.14	3.75	2.04
**P_4_PEG_**	(101)	26.56	0.236	36.09	2.43	0.76

**Table 4:** Surface energy of TiO_2_ film as a function of dispersants added in sol–gel solution.

Samples	Contact Angle, (°)			Surface Energy, (mJ m^2^)
	DI Water	EG	DMSO	
**P_4_**	26 ± 0.67	17 ± 0.24	29 ± 0.14	67
**P_4_** **___** **_PEG_**	16 ± 0.21	5 ± 0.84	3 ± 0.60	71

**Table 5:** Electric parameters from fitting experimental EIS data.

Samples	Electric Parameters			
	R_s_ (Ω cm^2^)	R_ct_ (Ω cm^2^)	CPE (Ω^−1^ cm^−2^ s^n^)	n
**P_4_**	21.60	418.0 × 10^3^	11.08 × 10^−6^	0.803
**P_4_PEG_**	15.50	23.4 × 10^3^	2.48 × 10^−3^	0.975

## Data Availability

The raw/processed data generated in this work are available upon request from the corresponding author.
